# Near-patient tests and the clinical gaze in decision-making of Swedish GPs not following current guidelines for sore throat – a qualitative interview study

**DOI:** 10.1186/s12875-015-0285-y

**Published:** 2015-07-04

**Authors:** Hedvig Gröndal, Katarina Hedin, Eva Lena Strandberg, Malin André, Annika Brorsson

**Affiliations:** Department of Sociology, Uppsala University, Uppsala, Sweden; Department of Clinical Sciences, Malmö, Family Medicine, Lund University, Lund, Sweden; Department of Research and Development, Region Kronoberg, Växjö, Sweden; Blekinge Centre of Competence, Blekinge County Council, Karlskrona, Sweden; Department of Medicine and Health Sciences, Family Medicine, Linköping University, Linköping, Sweden; Department of Public Health and Caring Sciences – Family Medicine and Preventive Medicine, Uppsala University, Uppsala, Sweden; Center for Primary Health Care Research, Skåne Region, Malmö, Sweden

**Keywords:** Near-patient tests, Sore throat, Guidelines, Decision-making, Qualitative interview study

## Abstract

**Background:**

Excessive antibiotics use increases the risk of resistance. Previous studies have shown that the Centor score combined with Rapid Antigen Detection Test (RADT) for Group A Streptococci can reduce unnecessary antibiotic prescribing in patients with sore throat. According to the former Swedish guidelines RADT was recommended with 2–4 Centor criteria present and antibiotics were recommended if the test was positive. C- reactive protein (CRP) was not recommended for sore throats. Inappropriate use of RADT and CRP has been reported in several studies.

**Methods:**

From a larger project 16 general practitioners (GPs) who stated management of sore throats not according to the guidelines were identified. Half-hour long semi-structured interviews were conducted. The topics were the management of sore throats and the use of near-patient tests. Qualitative content analysis was used.

**Results:**

The use of the near-patient test interplayed with the clinical assessment and the perception that all infections caused by bacteria should be treated with antibiotics. The GPs expressed a belief that the clinical picture was sufficient for diagnosis in typical cases. RADT was not believed to be relevant since it detects only one bacterium, while CRP was considered as a reliable numerical measure of bacterial infection.

**Conclusions:**

Inappropriate use of near-patient test can partly be understood as remnants of outdated knowledge. When new guidelines are introduced the differences between them and the former need to be discussed more explicitly.

## Background

Excessive use of antibiotics is the main cause of the increasing resistance [1]. Evidence-based guidelines are a strategy to reduce unnecessary antibiotic use. Previously all micro-organisms were considered equally qualifying for treatment with antibiotics while current guidelines explicitly state when patients benefit from antibiotic treatment [2]. A strategy frequently advocated to reduce diagnostic uncertainty and antibiotic prescription includes the use of near-patient tests [3].

In most countries guidelines recommend using the Centor score when diagnosing a sore throat [4]. This score (absence of cough, fever ≥38.5 degrees Celsius, tender lymphadenitis and tonsillar coating) predicts the likelihood of finding Streptococcus group A (GAS) [5]. Some guidelines also recommend using Rapid Antigen Detection Tests (RADT) for GAS In several countries pharyngotonsillitis is considered a self-limiting disease, where the risk of serious complications is so small that antibiotic treatment is seldom needed. In these countries the use of RADT is very modest [4].

According to the Swedish guidelines for pharyngotonsillitis applying when this study was conducted, RADT was recommended when 2–4 Centor criteria were present and antibiotics were recommended when the test was positive. The near-patient test, C-reactive protein (CRP) was not recommended [6].

Several studies show a substantial decrease in antibiotic prescribing after the introduction of RADT [7, 8]. However, use of RADT not in adherence with guidelines and hence unnecessary antibiotic prescribing has also been reported from countries where the test is used [8–11].

Near-patient CRP tests have been demonstrated to decrease unnecessary antibiotic use in lower respiratory tract infections, but evidence for diagnostic benefit in patients with sore throat is limited [12]. Despite this a frequent use has been reported in patients with sore throat in Sweden [10, 13]. In Swedish Primary Health Care, GPs have used the near-patient RADT and CRP for approximately 25 years without personal responsibility for laboratory costs. The tests are frequently used sometimes even before clinical assessment. CRP is reported to be used for around 30 % of the patients with respiratory tract infections and RADT for around 50 % of the patients with sore throat [10].

Little is known about GPs’ reasons for using near-patient tests and their relation to antibiotic prescription in patients with sore throat, and there have been calls for research on this [8]. Factors discussed in previous qualitative studies influencing GPs’ prescribing of antibiotics for respiratory tract infections are mainly related to the GPs’ perceptions [14]. In this study we draw on the understanding of medical technologies presented in the field of science and technology studies (STS), where medical technologies and guidelines are perceived as active parts in shaping the definition of health and illness, [15] and thus we examine how near-patient tests influence the management of sore throat and related prescribing of antibiotics.

In a previous study, we gave a general description of the actions and difficulties reported by GPs concerning the management of patients with sore throat in relation to guidelines [16]. A majority of the GPs showed significant knowledge gaps and could not recall the Centor criteria. GPs non-adherent to guidelines expressed a belief that any bacterial infection should be identified and treated with antibiotics without reference to treatment benefit. The aim of the present study was to deepen the understanding of what role the near-patient tests play in the decision- making of these GPs who do not follow guidelines in their management of patients with sore throat.

## Methods

This study was part of a larger project with a qualitative design, aimed to understand how GPs manage patients with a sore throat. Initially a strategic sample of 25 GPs was chosen with regard to sex, age, educational background, working experience, urban or rural Primary Health Care Centres (PHCC) as well as areas with high and low antibiotic prescribing from five different counties in Sweden [16]. Among the 25 participating GPs, 16 GPs stated that their management did not adhere to current guidelines. Non-adherent GPs were identified as those who could not correctly recapture the four Centor criteria, did not state use of RADT for GAS when ≥ 2 Centor criteria were present and did not state a positive RADT as a prerequisite for antibiotic treatment. This study was based on the subgroup of these 16 GPs.

The data were collected through individual semi-structured interviews with open-ended questions. Topics for the interviews were description of the management of patients with sore throat and use of near-patient tests. Four of the authors conducted the half-hour long interviews in the summer and early autumn of 2012 in a place chosen by the interviewed GP.

The interviews were audio-recorded and transcribed verbatim by a secretary. To ensure consistency the interviewers read each other’s interviews continuously. All but one of the authors had previously been involved in implementing sore throat guidelines, nationally or locally.

Qualitative content analysis guided by systematic text condensation according to Malterud was used [17]. To maximise theoretical sensitivity and rigour, all authors read the transcripts independently to get an overview. In the next step we identified and coded the meaning units representing different aspects of the participants’ experiences. Then the codes were organised into categories and themes by all the authors in an iterative process throughout the analysis until consensus was reached. The analysis was performed manually.

According to Swedish legislation, ethical approval from the regional ethics committee was not necessary due to the character of the study as part of a quality improvement activity. The study was, however, approved by a local ethics committee (Kronoberg ethics committee 8/2012). All participants gave their informed consent and were informed that participation was voluntary and that they could withdraw at any time, that all data was handled confidentially and that the results would be presented in a non-identifiable way.

## Results

The background characteristics of the GPs non-adherent to guidelines for sore throat are presented in Table 1. The analysis of the interviews revealed interplay between the perception that all infections caused by bacteria should be identified and prescribed antibiotics and the use of the clinical assessment and near-patient tests. Different management strategies were identified among the GPs non-adherent to guidelines (Table 2). Most GPs stated that they used more than one strategy.

**Table 1:** Description of the 16 participants

Category	Variable	Number of participants
Gender	Female	10
Age	≥45	12
Medical education	In Sweden	11
Working in primary health care	≥15 years	6
Employment status	Temporary pool physician	2
	GP trainee	2
	GP	12
Location of practice	City	2
	Town	10
	Village	4
Publicly run		12
Antibiotic prescription level in the county	High level	5
	Medium level	5
	Low level	6

**Table 2:** The clinical assessment, near-patient Rapid Antigen Detection Tests (RADT) and C-reactive protein (CRP)

Quotation	Code	Category
Quotation A	No RADT when typical picture	Clinicalpicture makes RADT unnecessary in typical cases but used when in doubt
If they then have what’s typical for me, that they have a swollen throat with a really, you know, nasty throat, and lymph glands on the throat and just throat symptoms and fever, then I tend to think like this, yes, this is classic tonsillitis, then I don’t take any tests. (Interview 2, p. 2)		
Quotation B	No RADT when decision to treat	
Then I don’t send them to get Strep-A either, if I am going to treat them, unless it is of some significance. Interview 11, p. 7		
Quotation C	RADT only when in doubt	
But sometimes I’m uncertain, and then I take and I see that tonsillitis is… the patient has enlarged tonsils and redness, but if there’s no furring or anything, then I can take Strep-A. (Interview 22, p. 5)		
Quotation D	Antibiotics when typical picture even if RADT is negative	Clinical picture dominates over negative RADT
A: Yes, yes, but even if Strep-A is negative, you sometimes give antibiotics, then?		
B: Yes.		
A: And what makes you give antibiotics all the same?		
B: If there is clear furring … and a high temperature and clear swelling and redness even though the Strep-A is negative, then I usually check monospot too if it has been longer than five to seven days. (Interview 14, p. 9)		
Quotation E	RADT does not show other bacteria	
B: Yes, obviously, if the patient is affected, it can be some other streptococcus than group A, and is affected and the like, then I can prescribe treatment. Interview 19, pp. 2–3		
Quotation F		
There are other types of streptococcus and other types of … kinds of bacteria. Interview 11, p. 12		
Quotation G	CRP indicates bacterial infection	CRP dominates over negative RADT
Then I take CRP too, to know whether it’s over fifty or sixty, then you think it’s something more bacterial than virus. (Interview 22, pp. 7–8)		
B: I usually go by zero to eight, normal, eight to seventy-five indicates that you have a virus infection and seventy-five to two hundred means bacterial. (Interview 19, pp. 6–7)		
Quotation H	Greater trust in CRP	
A: But if I understand you right, if Strep-A is negative you take CRP too?		
B: Exactly, yes, yes.		
A: And if the CRP is high, you treat?		
B: Yes. (Interview 5, pp. 6–7)		

First: The clinical presentation seemed to override RADT, making RADT for GAS unnecessary when patients presented a “typical” picture and RADT was used only when in doubt. Second: RADT, a test that detects one bacterium, GAS, was not considered reliable when negative. Third: Greater trust was placed in CRP which was used to determine whether the patient’s infection was caused by bacteria or viruses and seemed to override a negative RADT or was used instead of RADT. Fourth: The interaction between the clinical assessment and near patient-tests included several paths to the notion of bacterial infection that needed to be treated with antibiotics.

### Clinical picture makes RADT unnecessary in typical cases but was used when in doubt

The first and most frequent strategy was to prescribe antibiotics without using RADT when the clinical picture was perceived as “typical” for GAS tonsillitis. Arguments for this were that it is usually obvious if a patient has a GAS tonsillitis (Quotation A), or that the test would be confusing if the GP already had decided to treat(Quotation B). Consequently, these physicians reported using RADT only when in doubt or when the picture was not typical for tonsillitis (Quotation C).

### Clinical picture dominates over negative RADT

Another strategy was to use the test also when the clinical picture was perceived as typical. However, the clinical presentations sometimes took precedence in the decision when the result was negative. The arguments for antibiotic prescribing despite a negative result were similar to the arguments for not taking the test at all. The arguments were that the symptoms were typical (Quotation D) or that the infection could be caused by other types of bacteria than GAS which the test could not detect (Quotations E and F). The GPs frequently prescribed antibiotics immediately, but sometimes a negative result was followed by an additional test, CRP.

### CRP dominates over negative RADT

All GPs but one reported using CRP in patients with sore throat. CRP was used to determine whether the patient’s infection was caused by bacteria or viruses and if the result indicated bacteria antibiotics were prescribed (quotation G). Half of the GPs stated their threshold used for determining a “bacterial infection”. These varied between 40 and 200, but for a majority the threshold was 50. Several used CRP when the RADT was negative and when the test result was abnormal/elevated antibiotics were prescribed (Quotation H).

### Direct use of CRP

Half of the GPs said that they used the CRP test when the patients seemed very ill and had a fever and half of them said that they used CRP regardless of the patients’ condition (Table 3) (Quotation I).

**Table 3:** Direct use of C-reactive protein (CRP)

Quotation I	When the patient seems ill
A: Do you test CRP in these patients?	
B: Not necessarily and not everybody or many, so to speak, but … I would probably say that if it’s a patient with a generally affected condition, if it’s someone who seems really ill, then I probably test CRP too. (Interview 9, pp. 8–9)	
Quotation J	In general
A: Does it happen that you test CRP?	
B: Yes … often in advance, but even if I think that the patient is seriously ill, so to speak, or generally affected or sick and with fever, if you think of a sore throat, then yes. (interview 20, pp. 6–7)	

Moreover, some doctors stated that they frequently used CRP as a first step in the management of patients with sore throat, instead of, or at the same time as, RADT (Quotation J).

### Several paths to the notion of “bacterial infection”

**Fig. 1:**
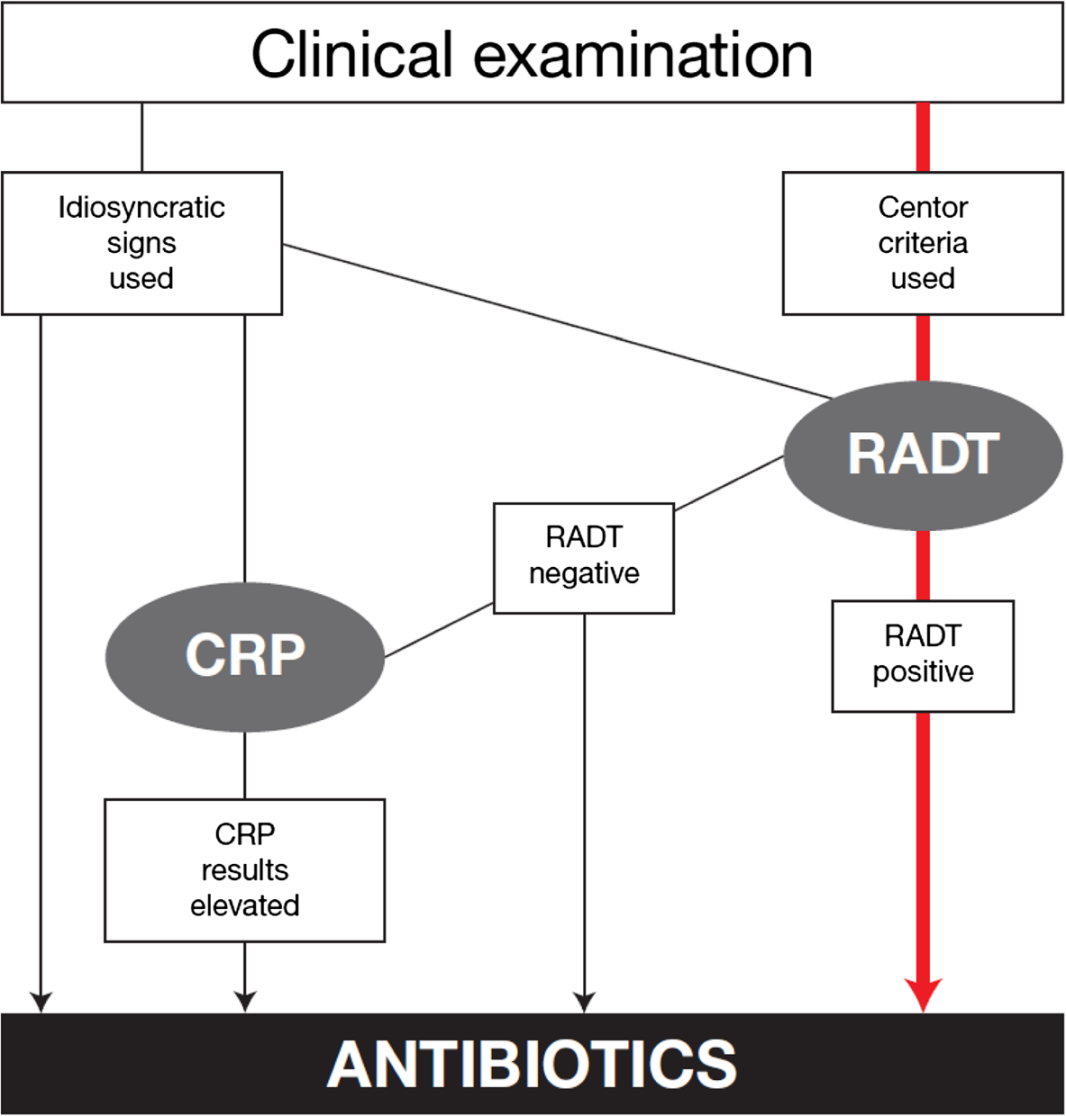
Different paths to antibiotic prescribing for sore throat in primary health care. The red arrow shows the diagnostic process according to guidelines. The black arrows show different deviations from guidelines reported by the participating GPs

The strategies reported by the GPs showed different alternatives deviating from guidelines for handling patients with sore throat. The clinical assessment and the laboratory tests seemed to interact in an intricate manner, which meant that there were several ways to end up in the notion of bacterial infection. These strategies are presented in a scheme where the different paths to bacterial infection – and thus antibiotics – become apparent (Fig. 1).

## Discussion

### Main findings

This study of GPs non-adherent to guidelines for sore throat revealed an interplay of the perception that all infections caused by bacteria should be identified and prescribed antibiotics with the use of the clinical gaze and near-patient tests. The clinical gaze seemed to override RADT: GAS infections were supposed to be visible and diagnosed by the naked eye. Instead RADT for GAS was used when in doubt. RADT, a test that detects one bacterium, GAS, was not considered reliable when negative. Moreover, greater trust was placed in CRP, which was used to determine whether the patient’s infection was caused by bacteria or viruses. CRP seemed to override a negative RADT or was used instead of RADT. The interaction between the clinical assessment and near patient-tests entailed several paths to the notion of bacterial infection that needed to be treated with antibiotics.

### Strengths and limitations

The strength of the study is the focus on the detailed questions which give a more profound and detailed understanding than previous studies [7–11, 13, 14]. Another strength of the study is the maximum variety sampling of participants from different parts of Sweden. Also, none of invited GPs declined to be interviewed. Moreover, all authors read all the interviews and participated in the analysis until consensus was reached.

This study has some weaknesses. Four different interviewers may have decreased the reliability of the interviews. This was counteracted by the use of the same interview guide and the continuous reading of each other’s transcripts to reach consistency. This process may have added different perspectives and provided more depth to the data. The fact that all but one of the interviewers had been involved in implementing the guidelines earlier may have biased the interviews, but this experience may also have contributed to the relevance of the interview guide. Another weakness is that this study does not include the actual use of near-patient tests by the GPs interviewed, since the link between statements and actual management is not always straightforward.

### Comparison with existing literature

The present study shows that use of the clinical assessment and near-patient test interplayed with the notion that bacterial infection needs to be treated with antibiotics in an intricate way. Our results thus deepen the understanding of results from earlier quantitative studies showing antibiotic prescribing without testing and despite negative test as well as use of RADT in patients with few Centor criteria [7–11].

The most frequently stated strategy among the interviewed GPs was to prescribe antibiotics without testing when the picture was “typical”. However, the interviewed GPs seemed not aware of the Centor criteria and used idiosyncratic signs. Thus the GPs seemed to believe that they could detect bacterial infection by the naked eye. This can be discussed as a remnant of previous practice when RADT was not used. Internationally this is still the case; a recent qualitative study from the UK concludes that it is unlikely that RADTs will be used in common practice in the near future [18]. The GPs’ confidence in their clinical gaze has connections to Foucault’s study of modern medicine in the late 1700s where the medical gaze was described as a new way to look at the patient. The clinician became the reader of the sick organism, searching for the cause of illness behind the surface of the body. Signs indicating disease were given priority and were separated from the patient as a whole [19]. The present study shows that these GPs still seem to rely more on their senses and own assessment, than on laboratory tests, −the clinical gaze is their main tool when handling these patients. When doing so, the aim is to find underlying bacteria, which can be seen as an example of Foucault’s medical gaze.

The most common explanation for treating a patient with antibiotics despite negative RADT was that the relevance of the test result was questioned. The first impression of the patient’s degree of illness outweighed the result of the test, in line with an early British study, where the GPs did not change their first decision to prescribe antibiotics when they got a negative RADT. The authors concluded that the time was not right for the test [20]. However, these results have been reproduced in several later studies [8, 9]. The strategy of ignoring the test result or not using the test at all might be explained by the notion that one diagnostic technique often dominates in defining a disease in order to avoid incompatible results [20]. In this case the clinical picture seemed to outweigh the negative test result of RADT. Moreover, as RADT detects only one bacterium, GAS, it was not considered useful for the task of identifying any bacterial infection.

RADT was used in cased of uncertainty. Near-patient tests, such as RADT and CRP, are appreciated by GPs to decrease diagnostic uncertainty as well as to enhance the communication with the patient [3]. However, when RADT is used in patients with low probability of GAS, the positive predictive value of the test will decrease and the percentage of false positive increase [5]. Moreover, the carriers may test positive and unnecessary antibiotic prescribing may increase.

CRP was appreciated for its universal applicability in distinguishing between bacteria and viruses, while RADT which is specific for GAS was considered inferior. The near-patient CRP was introduced in primary care to differentiate bacterial from viral infections [21]. Two small studies report CRP to have a discriminative value in throat infections caused by bacteria, Streptococcus group A [21] besides Streptococcus group C and *Hemophilus influenzae*, [12] but no studies have been found showing the advantage of using CRP to identify patients with sore throat who benefit from antibiotics.

From a science and technology studies perspective, medical technologies such as CRP intervene in the situations where they are used [15]. In the case of CRP the test results are presented numerically, resembling an interval scale. This seems to create an image of a measurable bacterial threat valid for all kinds of bacteria. Thus, the test seems to strengthen the conception that it is both possible and desirable to detect and treat bacteria above GAS and that the aetiological agent should be prioritised above treatment of the symptoms. This is in line with several descriptions of modern medical reasoning; the purpose of the clinician is to discover and treat an underlying pathology, thus searching for the cause of disease [22]. The use of CRP may decrease the uncertainty of the GP but builds on outdated, not evidence-based, knowledge. However, use of RADT not according to guidelines is reported from many countries where CRP is not used in primary care [7–9]. Therefore CRP alone cannot explain this practice, yet this study indicates that the use of CRP in patients with a sore throat maintains this outdated knowledge.

New guidelines aim to change practices, but in order to achieve that, they have to incorporate past procedures and routines, thus negotiations with pre-existing practices are required [23]. Thus, in the setting studied the negotiations take place in a transition from former guidelines and practice, which convey that bacteria should be identified and treated with antibiotics, to new guidelines and practice which imply that antibiotics should only be used when there is evidence of advantages that exceed the risks. Current Swedish guidelines also state that the aim of treatment is to reduce symptoms – thus putting the symptoms instead of the cause of infection in focus [6]. At the same time, however, they recommend the use RADT based on the Centor criteria. Although the Centor criteria are quite specific, the post-test probability of GAS pharyngitis is relatively low, and therefore the presence of GAS should be confirmed by RADT [5].

## Conclusions

This interview study of GPs non-adherent to guidelines for sore throat showed that their decision making was based on the perception that all infections caused by bacteria should be identified and given antibiotics. They used their clinical gaze and near-patient tests according to this position.It is evident that guidelines have not permeated the profession despite the twelve years that have elapsed. When new guidelines and technologies are introduced to the profession, the differences between them and the previous ones should be addressed more explicitly. Moreover, unforeseen consequences of introducing new tests without proper prior assessment in the clinical everyday work should be taken into consideration. Further studies are needed to relate the reasoning of GPs to their actual management. These could preferably be conducted with a mixed methods approach.

### Availability of supporting data

All the supporting data are included as additional files.

## Abbreviations

CRP: C reactive protein
GAS: Group A Streptococcus
GP: General Practitioner
RADT: Rapid Antigen Detection Test
